# Supplementary material for: Atlas-scale single-cell multi-sample multi-condition data integration using scMerge2
Source: Nat Commun. 2023 Jul 17;14:4272. doi: 10.1038/s41467-023-39923-2 (PMC10352351; doi:10.1038/s41467-023-39923-2)
Supplement: Supplementary file 1 — Supplementary Information [file 41467_2023_39923_MOESM1_ESM.pdf]

Supplementary Information

Supplementary Information for "Atlas-scale single-cell multi-sample multi-condition data integration using scMerge2"

Table of content

A. Supplementary Figures S1-18

B. Supplementary Table 1: Data collections used in the paper.

Supplementary Figures

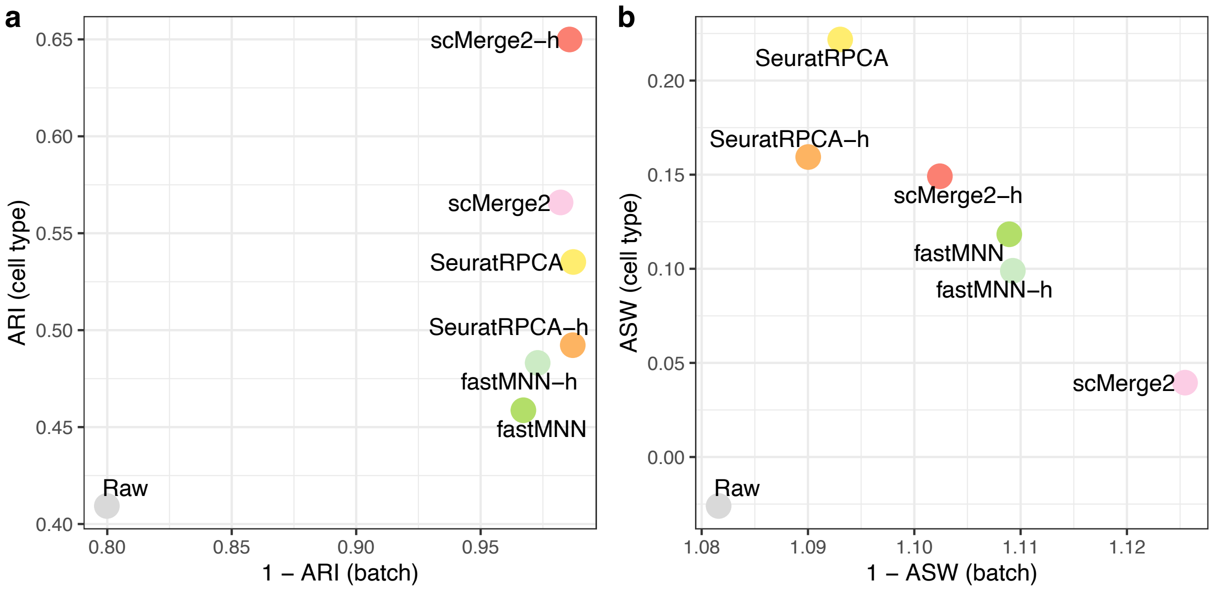

Supplementary Figure S1: Data integration evaluation to compared methods with hierarchical merging settings: Scatter plots of evaluation metrics of data integration of a 200k cells COVID-19 data for scMerge2, scMerge2-h, SeuratRPCA, SeuratRPCA-h, fastMNN and fastMNN-h: (a) Adjusted rand index (ARI) , where x-axis indicates 1 minus batch ARI and y-axis indicates cell type ARI; (b) Average silhouette width (ASW), where the x-axis is 1 minus batch ASW and y-axis is the cell type ASW.

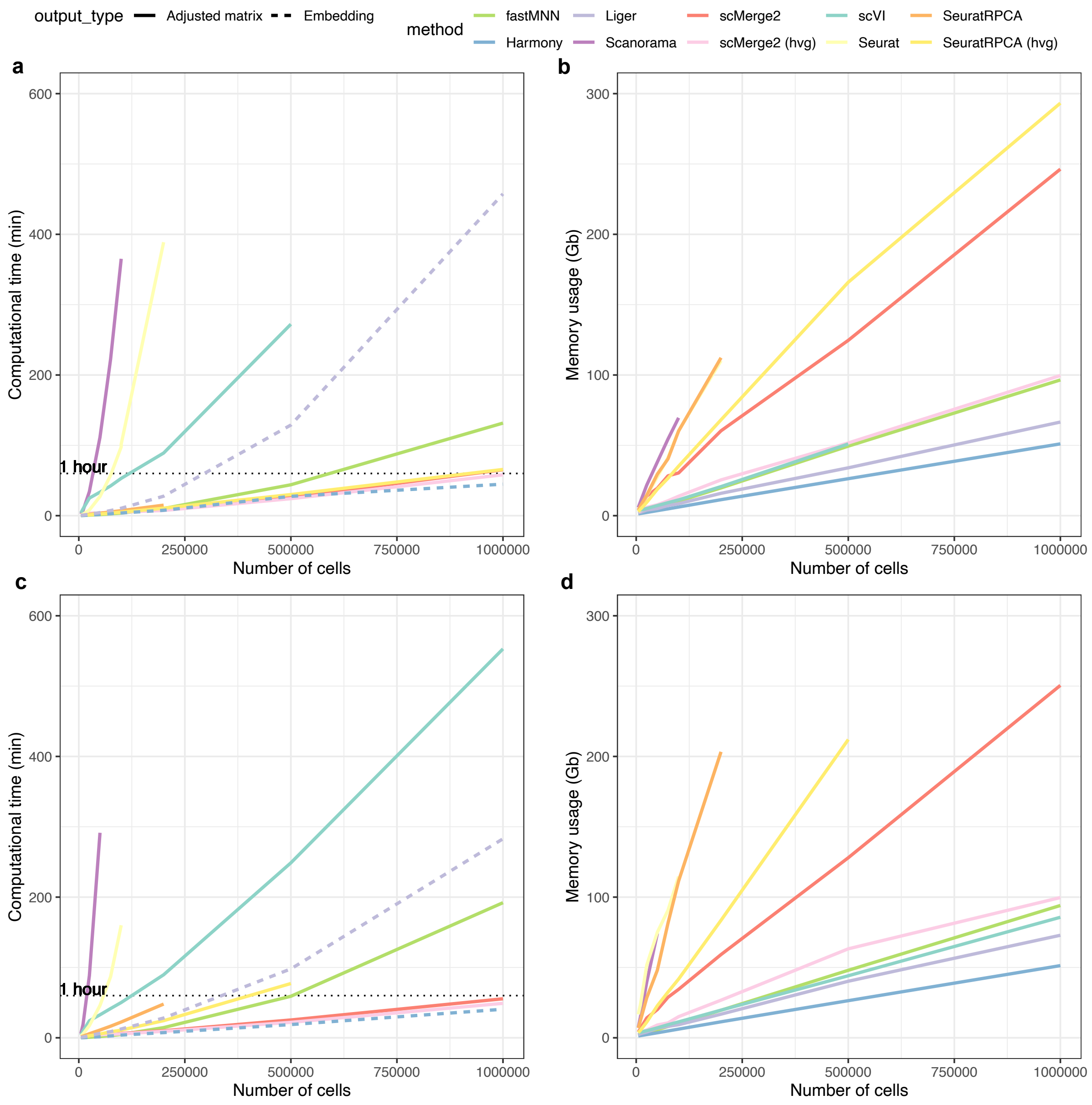

Supplementary Figure S2: Scalability of each data integration method of the RNA integration task with (a-b) 2 batches in the data and (c-d) 6 batches in the data, colored by the methods and the type of the lines indicate the output type of the methods. (a, c) shows computational time (min) for each method increase with the number of cells in the data; (b, d) shows memory usage (Gb) for each method increase with the number of cells. Note that SeuratRPCA encountered a memory error when integrating the full gene expression matrix of 500k cells, and SeuratRPCA (hvg) encountered a memory error when integrating the full gene expression matrix of 1 million cells for 6 batches cases.

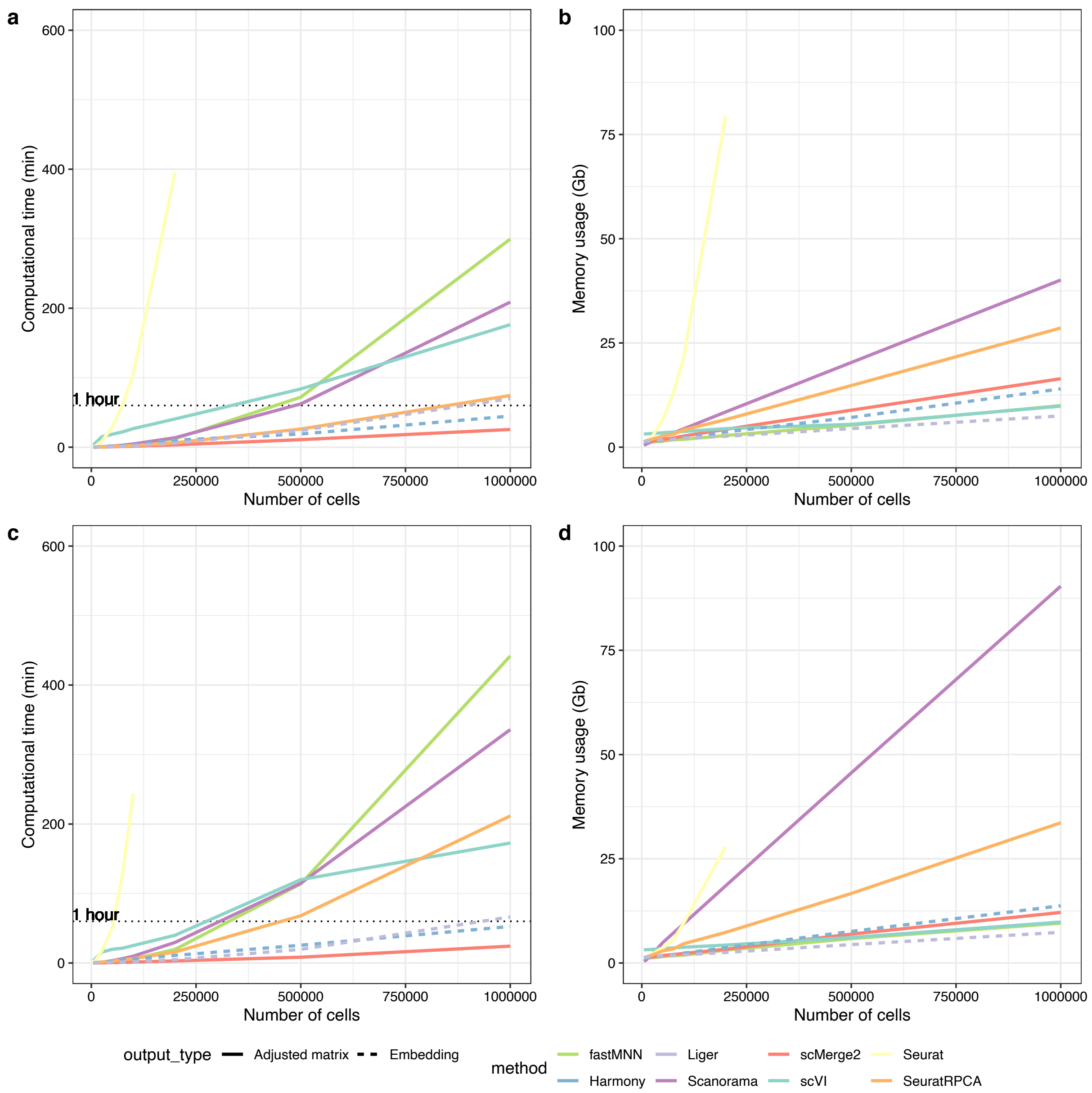

Supplementary Figure S3: Scalability of each data integration method of the ADT integration task with (a-b) 2 batches in the data and (c-d) 6 batches in the data, colored by the methods and the type of the lines indicate the output type of the methods. (a, c) shows computational time (min) for each method increase with the number of cells in the data; (b, d) shows memory usage (Gb) for each method increase with the number of cells.

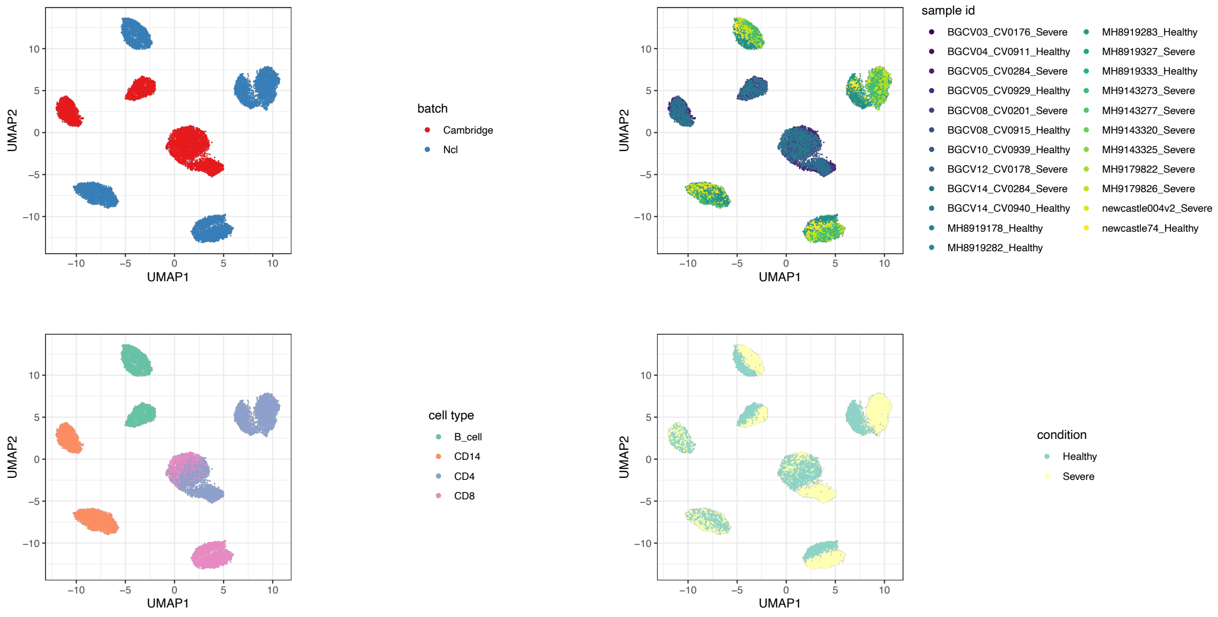

Supplementary Figure S4: UMAP plots of an example of simulated data (logFC = 1.2, DS% = 5%), coloured by batch, sample id, cell type and condition.

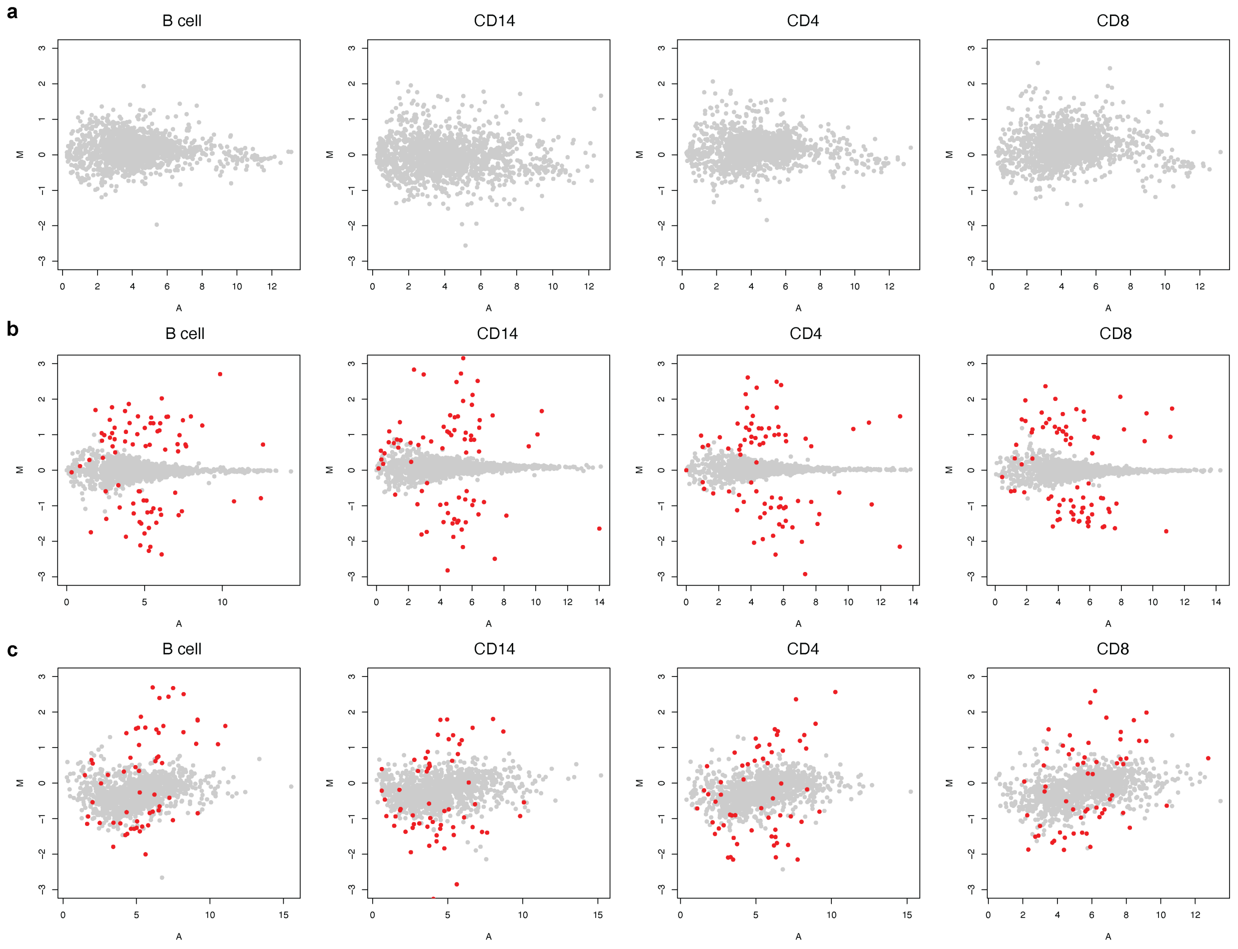

Supplementary Figure S5: MA plots of the real and simulated data, where x-axis is the average of gene expression and y-axis is the difference of the gene expression between two condition: (a) Real data; (b) Simulated data using mu formula ∼`cell type`, estimated from data with one condition; (c) Simulated data using mu formula ∼`cell type + sample ID + condition`, estimated from data from two conditions but with condition label permuted. The red dots indicates the simulated ground truth DS genes. The simulation strategy (c) exhibits a more similar pattern with the real data, which therefore is used in this study.

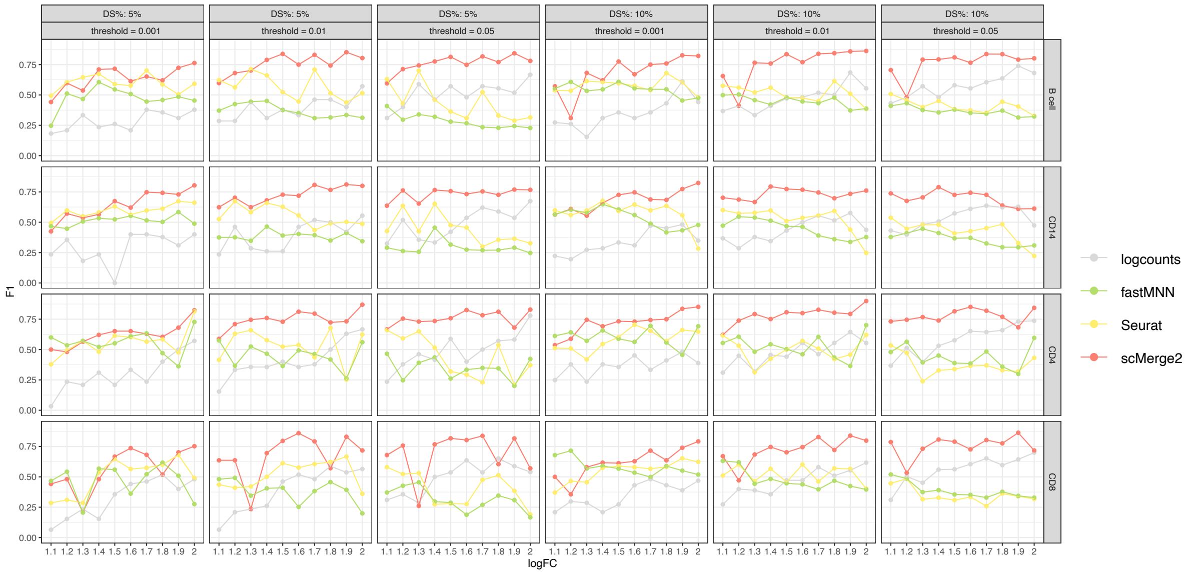

Supplementary Figure S6: F1-score of the differential state (DS) results of four cell types (B cell, CD14, CD4 and CD8) (row) of simulated data, with 5% (1st - 3rd column) and 10% DS genes (4th - 6th column) within each cell type, for scMerge2, Seurat, fastMNN and raw, varying simulated log fold change (logFC) of DS genes (x-axis) and different threshold of adjusted p-value (column).

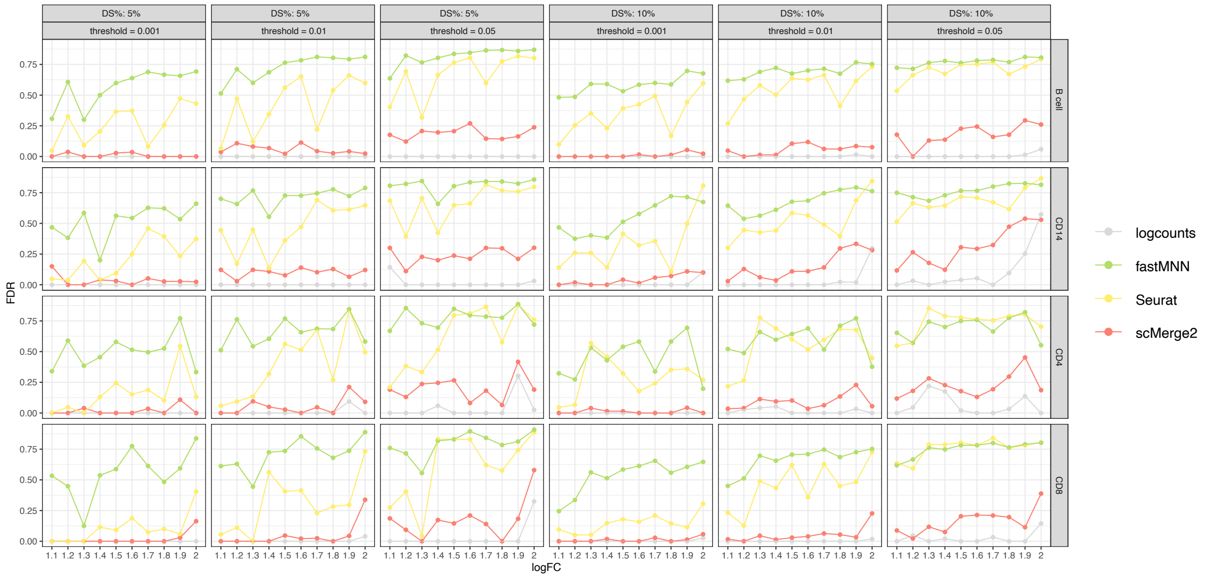

Supplementary Figure S7: FDR of the differential state (DS) results of four cell types (B cell, CD14, CD4 and CD8) (row) of simulated data, with 5% (1st - 3rd column) and 10% DS genes (4th - 6th column) within each cell type, for scMerge2, Seurat, fastMNN and raw, varying simulated log fold change (logFC) of DS genes (x-axis) and different threshold of adjusted p-value (column).

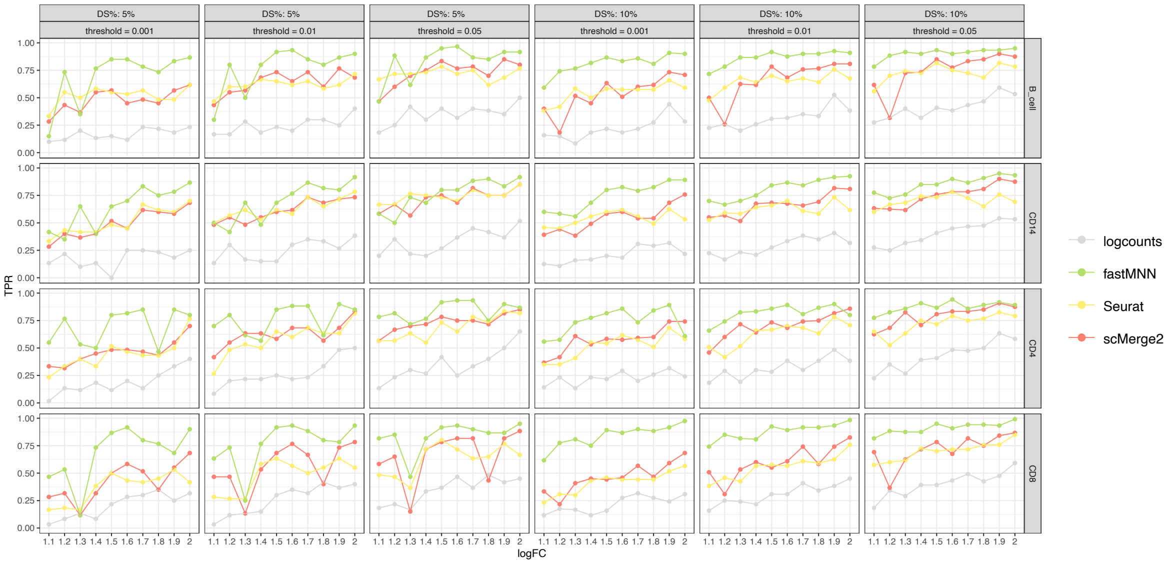

Supplementary Figure S8: TPR of the differential state (DS) results of four cell types (B cell, CD14, CD4 and CD8) (row) of simulated data, with 5% (1st - 3rd column) and 10% DS genes (4th - 6th column) within each cell type, for scMerge2, Seurat, fastMNN and raw, varying simulated log fold change (logFC) of DS genes (x-axis) and different threshold of adjusted p-value (column).

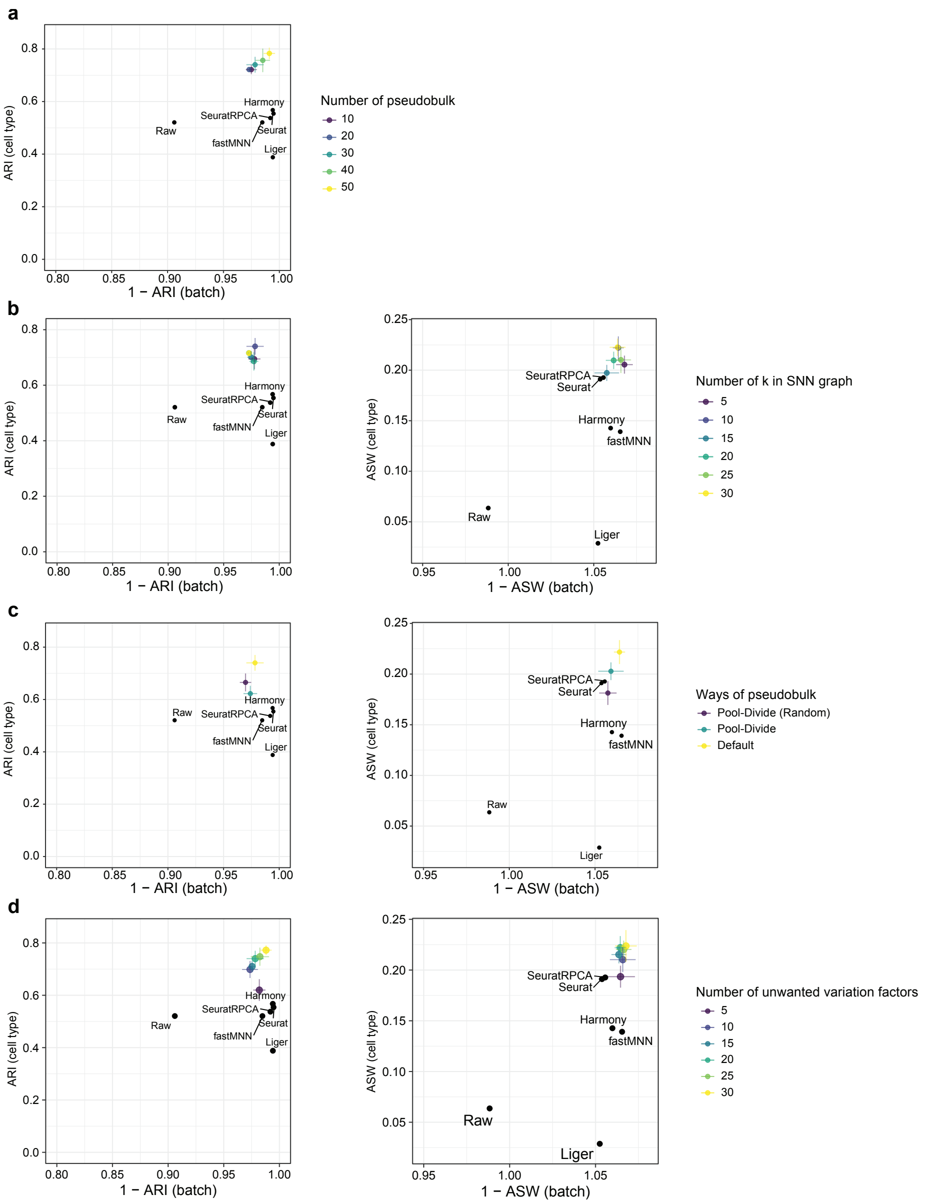

Supplementary Figure S9: Robustness analysis of the tuning parameters of scMerge2 using COVID-19 60k data: Adjusted rand index (ARI) (left panel), where x-axis indicates 1 minus batch ARI and y-axis indicates cell type ARI; Average silhouette width (ASW), where x-axis indicates 1 minus batch ASW and y-axis indicates cell type ASW (right panel), when varying (a) the number of pseudobulk constructed (10, 20, 30 (default), 40, 50); (b) the number of k used in SNN graph (5, 10 (default), 15, 20, 25, 30); (c) different methods to construct pseudobulk. (d) Number of unwatned variation factors (5, 10, 15, 20 (default), 25, 30).

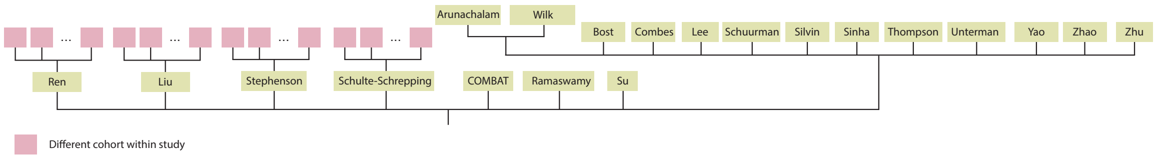

Supplementary Figure S10: Hierarchical merging strategy for COVID-19 scRNA-seq data collection.

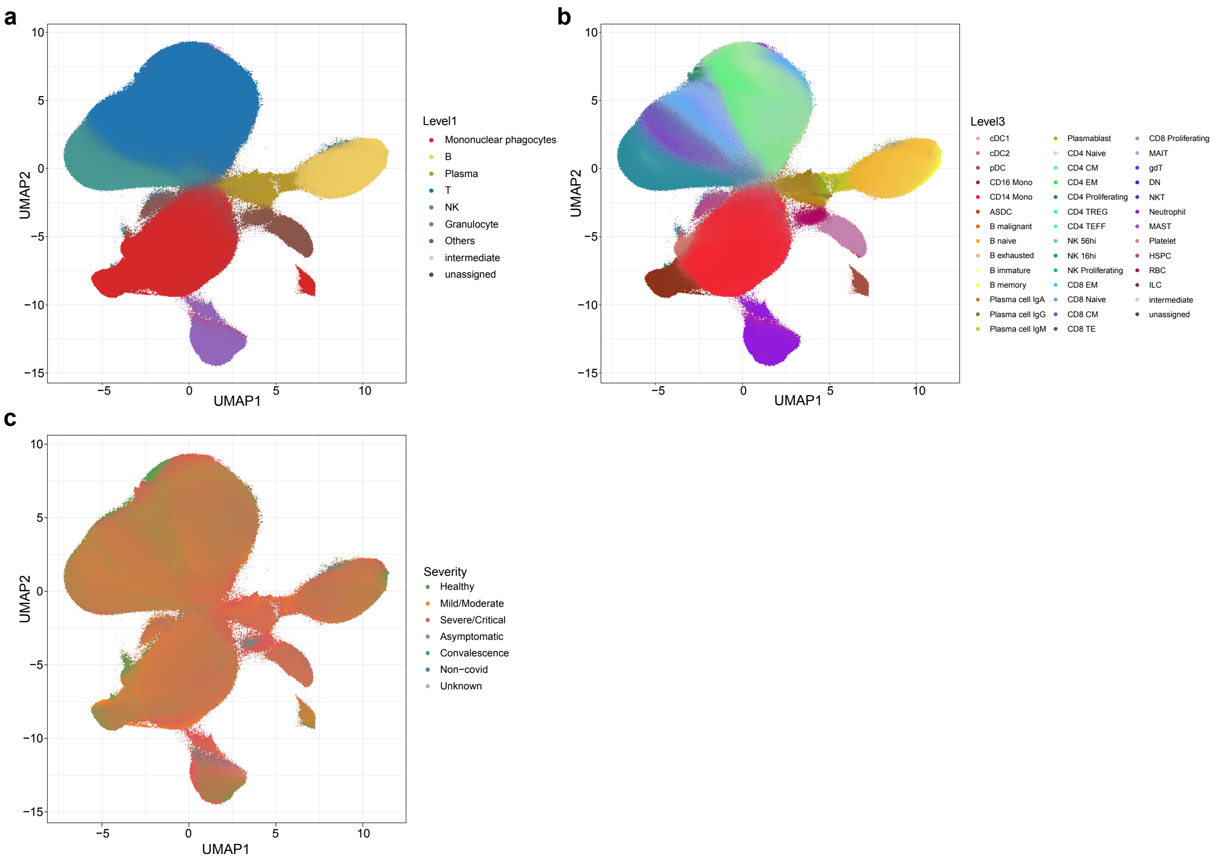

Supplementary Figure S11: UMAP of integration of COVID-19 data collection after scMerge2 integration, coloured by (a) level 1 cell type annotation; (b) level 3 cell type annotation and (c) severity.

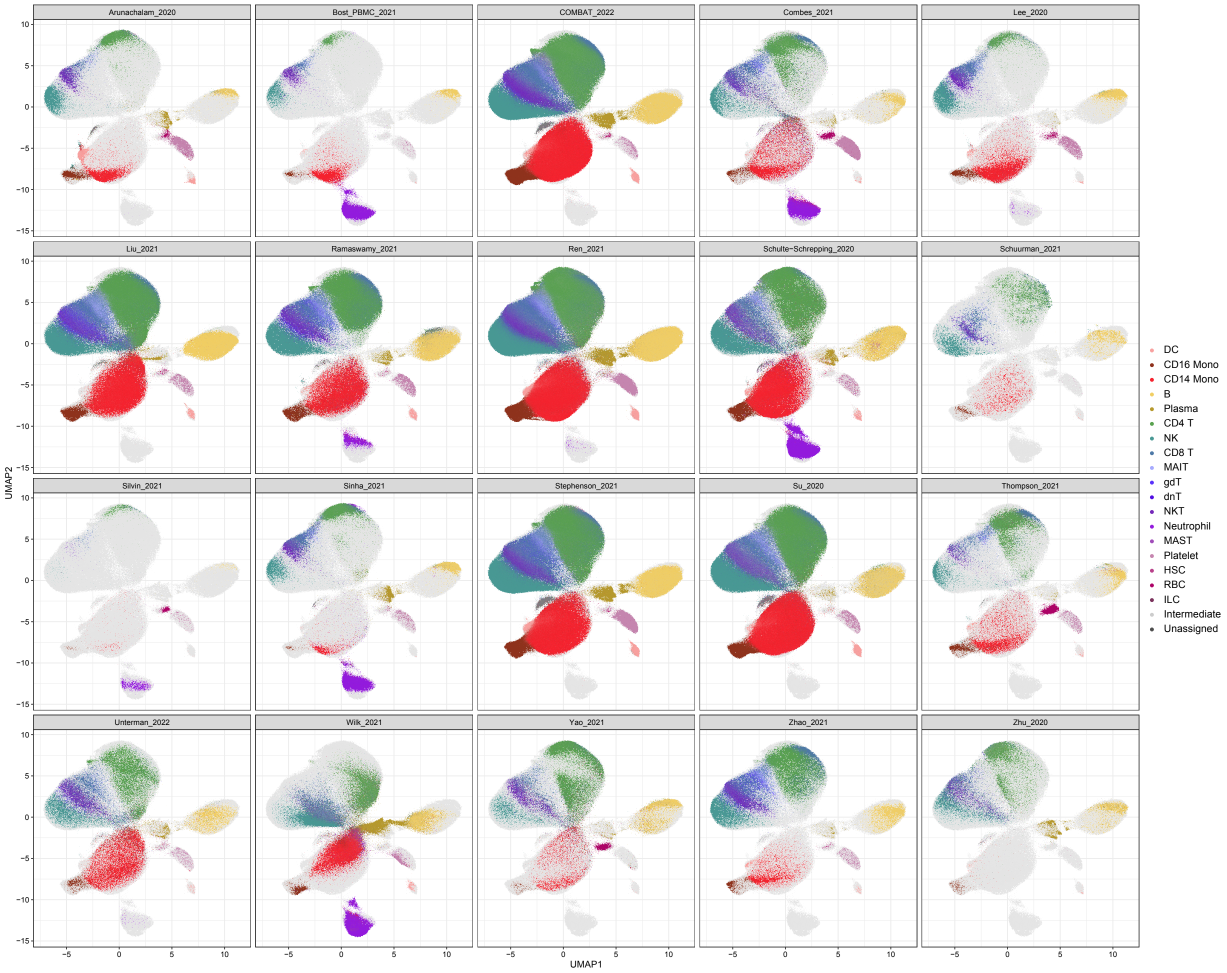

Supplementary Figure S12: UMAP of integration of COVID-19 data collection after scMerge2 integration, coloured by cell type (level 2) and faceted by dataset.

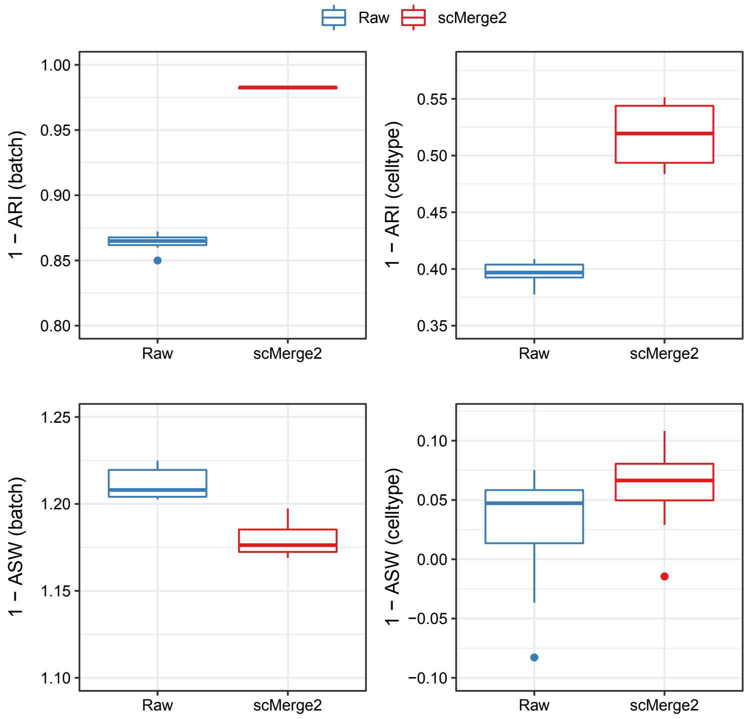

Supplementary Figure S13: Boxplots of evaluation metrics of COVID-19 scRNA-seq data collection for scMerge2-h (data merged in a hierarchical manner) and Raw, where the first row indicates the results of adjusted rand index (ARI): 1 minus batch ARI (left) and cell type ARI (right); the second row indicates the results of Average silhouette width (ASW): 1 minus batch ASW (left) and cell type ASW (right). For all of the four metrics, higher value indicates better performance. Since the size of this data collection is large, we subsampled 1% of the cells to calculate the metrics, and repeated this procedure 10 times. Each box ranges from the first to third quartile of evaluation metrics with the median as the horizontal line. The box plot's lower whisker extends 1.5 times the interquartile range below the first quartile, while the upper whisker extends 1.5 times the interquartile range above the third quartile.

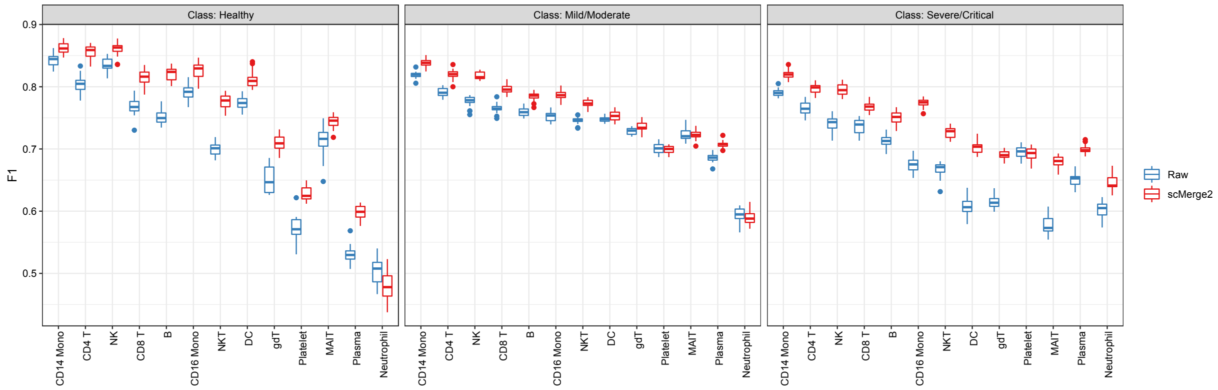

Supplementary Figure S14: Prediction results from 20 times repeated cross validation of disease severity using cell type-specific aggregated expression calculated from raw logcounts (blue) and scMerge2 adjusted results (red), evaluated by class-specific F1 scores. Each box includes 20 points, ranges from the first to third quartile of F1 scores with the median as the horizontal line. The box plot's lower whisker extends 1.5 times the interquartile range below the first quartile, while the upper whisker extends 1.5 times the interquartile range above the third quartile.

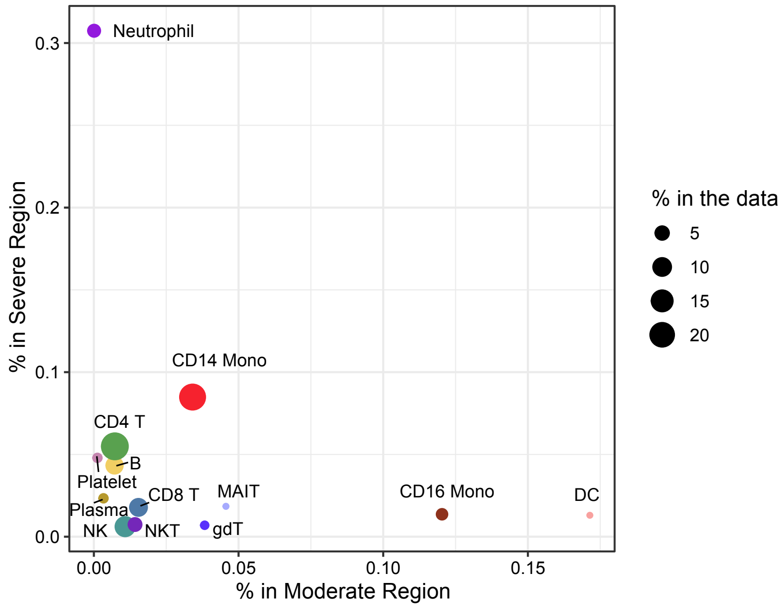

Supplementary Figure S15: Scatter plot shows the proportion of cells in Moderate region (x-axis) vs the proportion of cells in Severe region, determined by DAseq. The size of each point indicates the cell type proportion in the all data (Only cell types that have more than 1% in the data are shown).

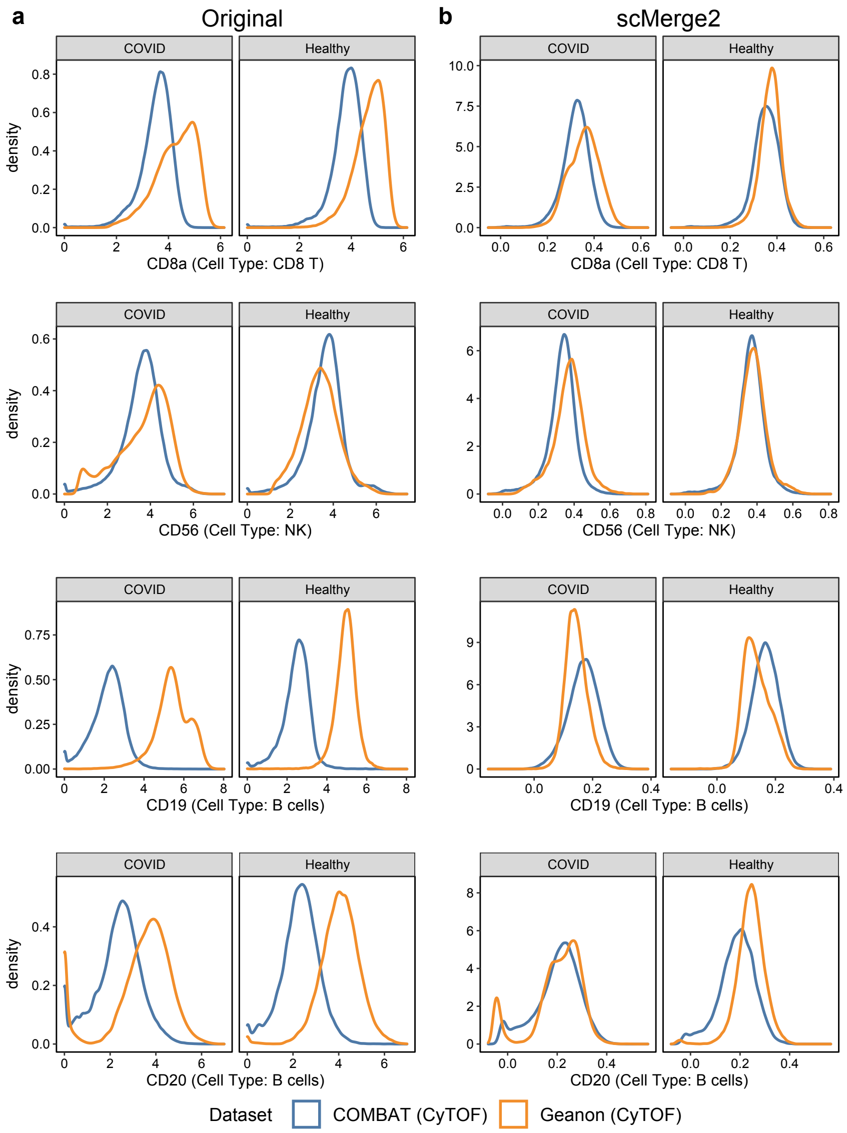

Supplementary Figure S16: Density plot of selected marker in specific cell type: CD8a in CD8 T cells; CD56 in NK cells; CD19 in B cells and CD20 in B cells, using (a) original expression and (b) scMerge2 adjusted expression. Within a specific cell type, the distribution of the cell type marker is expected to be similar between two datasets.

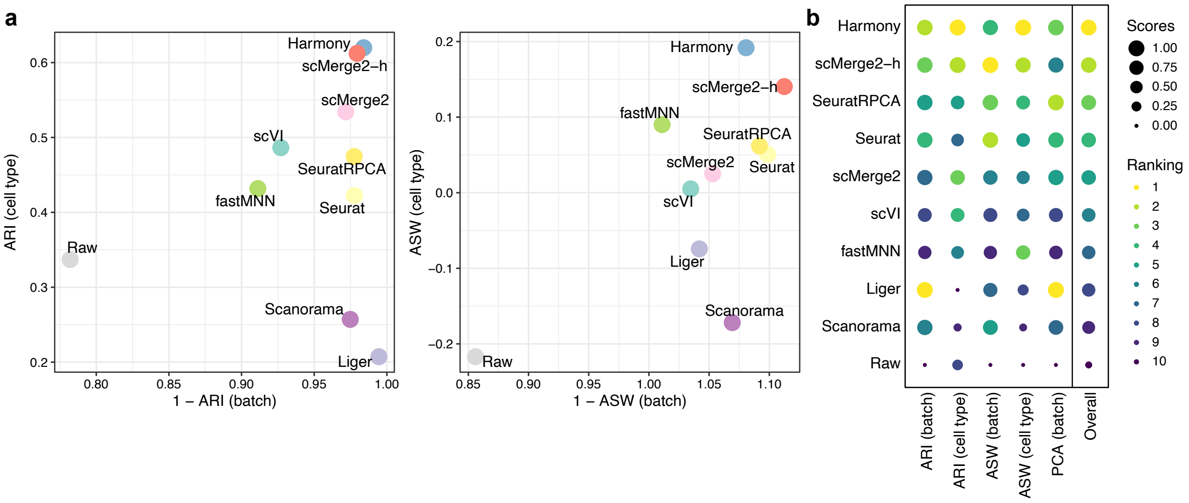

Supplementary Figure S17: CITE-seq data example: (a) Scatter plots of evaluation metrics of ADT data integration of a 200k cells subset of two COVID-19 studies (Liu and Stephenson) for scMerge2, scMerge2-h (data merged in a hierarchical manner), Seurat, Seurat (RPCA), Harmony, fastMNN, Liger, scVI, Scanorama and Raw: Adjusted rand index (ARI) (left panel), where x-axis indicates 1 minus batch ARI and y-axis indicates cell type ARI; Average silhouette width (ASW), where x-axis indicates 1 minus batch ASW and y-axis indicates cell type ASW (right panel). (b) Dot plots indicates the ranking of the data integration methods in terms of 5 different evaluation metrics. The size of the dot indicates the scaled scores, which are obtained from the min-max scaling of the original values. The overall ranking is ranked based on the average ranking of the five evaluation metrics.

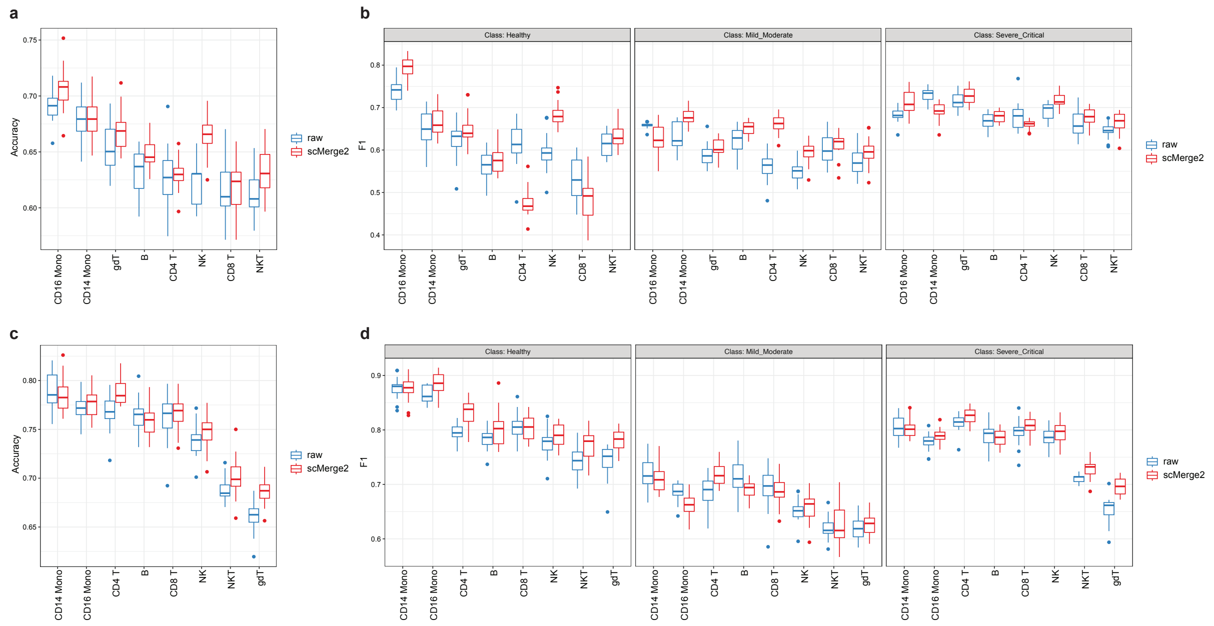

Supplementary Figure S18: CITE-seq data example: Prediction results from 20 times repeated cross validation of disease severity of disease severity using cell type-specific aggregated expression calculated from raw logcounts (blue) and scMerge2 normalised results (red), using (a-b) ADT expression and (c-d) RNA expression. Each box include 20 points, ranges from the first to third quartile of classification accuracy or F1 scores with the median as the horizontal line. The box plot's lower whisker extends 1.5 times the interquartile range below the first quartile, while the upper whisker extends 1.5 times the interquartile range above the third quartile.

Supplementary Table 1. Data collections used in the paper.

	Study	Accession	Number of cells	Number of samples	Number of donors	Country	Published Date	doi	Link to database
	Arunachalam_2020	GSE155673	56639	12	12	US	August 11, 2020	https://www.science.org/doi/10.1126/science.abc6261	https://www.ncbi.nlm.nih.gov/geo/query/acc.cgi?acc=GSE155673
	Bost_PBMC_2021	GSE157344	50284	33	33	Isreal	March 5, 2021	https://doi.org/10.1038/s41467-021-21702-6	https://www.ncbi.nlm.nih.gov/geo/query/acc.cgi?acc=GSE157344
	COMBAT_2022	EGAS00001005493	783704	140	140	UK	March 3, 2022	https://doi.org/10.1016/j.cell.2022.01.012	https://ega-archive.org/studies/EGAS00001005493
	Combes_2021	GSE163668	111990	44	44	US	January 25, 2021	https://doi.org/10.1038/s41586-021-03234-7	https://www.ncbi.nlm.nih.gov/geo/query/acc.cgi?acc=GSE163668
	Lee_2020	GSE147507	59572	20	17	Korea	July 10, 2020	https://www.science.org/doi/10.1126/sciimmunol.abd15544	https://www.ncbi.nlm.nih.gov/geo/query/acc.cgi?acc=GSE147507
	Liu_2021	GSE161918	411902	70	47	US	April 1, 2021	https://doi.org/10.1016/j.cell.2021.02.0188	https://www.ncbi.nlm.nih.gov/geo/query/acc.cgi?acc=GSE161918
	Ramaswamy_2021	GSE166489	271267	38	32	US	May 11, 2021	https://doi.org/10.1016/j.immuni.2021.04.003	https://www.ncbi.nlm.nih.gov/geo/query/acc.cgi?acc=GSE166489
	Ren_2021	GSE158055	999462	173	151	China	April 1, 2021	https://doi.org/10.1016/j.cell.2021.01.053	https://www.ncbi.nlm.nih.gov/geo/query/acc.cgi?acc=GSE158055
	Schulte-Schrepping_2020	EGAS00001004571	328780	147	74	Germany	September 17, 2020	https://doi.org/10.1016/j.cell.2020.08.001	https://ega-archive.org/studies/EGAS00001004571
	Schuurman_2021	GSE164948	32384	20	20	Netherlands	August 23, 2021	https://doi.org/10.7554/eLife.696611	https://www.ncbi.nlm.nih.gov/geo/query/acc.cgi?acc=GSE164948
**COVID-19 scRNA-seq**	Silvin_2020	E-MTAB-9221	6960	10	10	France	September 17, 2020	https://doi.org/10.1016/j.cell.2020.08.002	https://www.ebi.ac.uk/biostudies/arrayexpress/studies/E-MTAB-9221
	Sinha_2022	GSE157789	80994	21	14	Canada	January 1, 2022	https://doi.org/10.1038/s41591-021-01576-3	https://www.ncbi.nlm.nih.gov/geo/query/acc.cgi?acc=GSE157789
	Stephenson_2021	E-MTAB-10026	643071	143	130	UK	April 20, 2021	https://doi.org/10.1038/s41591-021-01329-2	https://www.ebi.ac.uk/biostudies/arrayexpress/studies/E-MTAB-10026
	Su_2020	E-MTAB-9357	538210	268	143	US	October 10, 2020	https://doi.org/10.1016/j.cell.2020.10.037	https://www.ebi.ac.uk/biostudies/arrayexpress/studies/E-MTAB-9357
	Thompson_2021	GSE166992	63895	8	8	US	March 16, 2021	https://doi.org/10.1016/j.celrep.2021.108863	https://www.ncbi.nlm.nih.gov/geo/query/acc.cgi?acc=GSE166992
	Unterman_2022	GSE155224	80789	18	10	US	January 22, 2022	https://doi.org/10.1038/s41467-021-27716-4	https://www.ncbi.nlm.nih.gov/geo/query/acc.cgi?acc=GSE155224
	Wilk_2021	GSE174072	174753	55	39	US	June 15, 2021	https://doi.org/10.1084/jem.20210582	https://www.ncbi.nlm.nih.gov/geo/query/acc.cgi?acc=GSE174072
	Yao_2021	GSE154567	69983	17	17	US	January 5, 2021	https://doi.org/10.1016/j.celrep.2020.108590	https://www.ncbi.nlm.nih.gov/geo/query/acc.cgi?acc=GSE154567
	Zhao_2021	https://figshare.com/articles/dataset/seu_obj_h5ad/16922467	88374	38	19	China	September 16, 2021	https://doi.org/10.1038/s41392-021-00753-7	https://figshare.com/articles/dataset/seu_obj_h5ad/16922467
	Zhu_2020	CNP0001102	46022	23	3	China	September 15, 2020	https://doi.org/10.1016/j.immuni.2020.07.009	https://db.cngb.org/search/project/CNP0001102/
		Total:	4899035	1298	963				
**COVID-19 CyTOF**	COMBAT_2022	EGAS00001005493	7118158	160	160	UK	March 3, 2022	https://doi.org/10.1016/j.cell.2022.01.012	https://ega-archive.org/studies/EGAS00001005493
	Geanon_2022	FR-FCM-Z2XA	4747543	21	21	US	February 16, 2021	https://doi.org/10.1002/cyto.a.24317	http://flowrepository.org/id/FR-FCM-Z2XA
		Total:	11865701	181	181				
**COVID-19 200k CITE-seq (COVID-19 200k)**	Liu_2021	GSE161918	82537	70	47	US	April 1, 2021	https://doi.org/10.1016/j.cell.2021.02.0188	https://www.ncbi.nlm.nih.gov/geo/query/acc.cgi?acc=GSE161918
	Stephenson_2021	E-MTAB-10026	117463	114	104	UK	April 20, 2021	https://doi.org/10.1038/s41591-021-01329-2	https://www.ebi.ac.uk/biostudies/arrayexpress/studies/E-MTAB-10026
		Total:	200000	184	151				
**COVID-19 60k**	Stephenson_2021	E-MTAB-10026	66967	58	53	UK	April 20, 2021	https://doi.org/10.1038/s41591-021-01329-2	https://www.ebi.ac.uk/biostudies/arrayexpress/studies/E-MTAB-10026
**COVID IMC**	Rendeiro_2021	zenodo.4110560,<br>zenodo.4139443,<br>zenodo.4637034	664006	237	23	US	March 29, 2021	https://doi.org/10.1038/s41586-021-03475-6	https://zenodo.org/record/4110560<br>https://zenodo.org/record/4139443<br>https://zenodo.org/record/4637034
