# Supplementary material for: Atlas-scale single-cell multi-sample multi-condition data integration using scMerge2
Source: Nat Commun. 2023 Jul 17;14:4272. doi: 10.1038/s41467-023-39923-2 (PMC10352351; doi:10.1038/s41467-023-39923-2)
Supplement: Supplementary file 3 — Reporting Summary [file 41467_2023_39923_MOESM3_ESM.pdf]

nature portfolio

Corresponding author(s): Jean Yang

Last updated by author(s): Jun 26, 2023

Reporting Summary

Nature Portfolio wishes to improve the reproducibility of the work that we publish. This form provides structure for consistency and transparency in reporting. For further information on Nature Portfolio policies, see our Editorial Policies and the Editorial Policy Checklist.

Statistics

For all statistical analyses, confirm that the following items are present in the figure legend, table legend, main text, or Methods section.

n/a	Confirmed	
☐	☒	The exact sample size (n) for each experimental group/condition, given as a discrete number and unit of measurement
☒	☐	A statement on whether measurements were taken from distinct samples or whether the same sample was measured repeatedly
☐	☒	The statistical test(s) used AND whether they are one- or two-sided<br>*Only common tests should be described solely by name; describe more complex techniques in the Methods section.*
☒	☐	A description of all covariates tested
☐	☒	A description of any assumptions or corrections, such as tests of normality and adjustment for multiple comparisons
☐	☒	A full description of the statistical parameters including central tendency (e.g. means) or other basic estimates (e.g. regression coefficient) AND variation (e.g. standard deviation) or associated estimates of uncertainty (e.g. confidence intervals)
☐	☒	For null hypothesis testing, the test statistic (e.g. F, t, r) with confidence intervals, effect sizes, degrees of freedom and P value noted<br>*Give P values as exact values whenever suitable.*
☒	☐	For Bayesian analysis, information on the choice of priors and Markov chain Monte Carlo settings
☒	☐	For hierarchical and complex designs, identification of the appropriate level for tests and full reporting of outcomes
☒	☐	Estimates of effect sizes (e.g. Cohen's d, Pearson's r), indicating how they were calculated

Our web collection on statistics for biologists contains articles on many of the points above.

Software and code

Policy information about availability of computer code

Data collection	No software was used during data collection.
Data analysis	Data analysis was performed in R (version 4.1.2), using the following packages: Seurat (v 4.1.1), batchelor (v1.12.3), rliger (v1.0.0), Harmony (v0.1.0), scVI (v0.16.1), Scanorama (v1.7.3), fgsea (v1.22.0), limma (3.50.3), edgeR (3.36.0). scMerge2 is implemented is part of the scMerge package (v1.15.0) (Github: https://github.com/SydneyBioX/scMerge).

For manuscripts utilizing custom algorithms or software that are central to the research but not yet described in published literature, software must be made available to editors and reviewers. We strongly encourage code deposition in a community repository (e.g. GitHub). See the Nature Portfolio guidelines for submitting code & software for further information.

Data

Policy information about availability of data

All manuscripts must include a data availability statement. This statement should provide the following information, where applicable:

- Accession codes, unique identifiers, or web links for publicly available datasets
- A description of any restrictions on data availability
- For clinical datasets or third party data, please ensure that the statement adheres to our policy

All the datasets used in this study are publcily available. All data used in this study are included in Supplementary Data 1.
The COVID-19 IMC data: The Arunachalam data used in this study is available in the GEO database under accession code GSE155673 (https://www.ncbi.nlm.nih.gov/

geo/query/acc.cgi?acc=GSE155673).
The Bost PBMC data is available in the GEO database under accession code GSE157344 (https://www.ncbi.nlm.nih.gov/geo/query/acc.cgi?acc=GSE157344).
The COMBAT data is available in the EGA database under accession code EGAS00001005493 (https://ega-archive.org/studies/EGAS00001005493).
The Combes data is available in the GEO database under accession code GSE163668 (https://www.ncbi.nlm.nih.gov/geo/query/acc.cgi?acc=GSE163668).
The Lee data is available in the GEO database under accession code GSE147507 (https://www.ncbi.nlm.nih.gov/geo/query/acc.cgi?acc=GSE147507).
The Liu data is available in the GEO database under accession code GSE161918 (https://www.ncbi.nlm.nih.gov/geo/query/acc.cgi?acc=GSE161918).
The Ramaswamy data is available in the GEO database under accession code GSE166489 (https://www.ncbi.nlm.nih.gov/geo/query/acc.cgi?acc=GSE166489).
The Ren data is available in the GEO database under accession code GSE158055 (https://www.ncbi.nlm.nih.gov/geo/query/acc.cgi?acc=GSE158055).
The Schulte-Schrepping data is available in the EGA database under accession code EGAS00001004571 (https://ega-archive.org/studies/EGAS00001004571).
The Schuurman data is available in the GEO database under accession code GSE164948 (https://www.ncbi.nlm.nih.gov/geo/query/acc.cgi?acc=GSE164948).
The Silvin data is available in the EBI database under accession code E-MTAB-9221 (https://www.ebi.ac.uk/biostudies/arrayexpress/studies/E-MTAB-9221).
The Sinha data is available in the GEO database under accession code GSE157789 (https://www.ncbi.nlm.nih.gov/geo/query/acc.cgi?acc=GSE157789).
The Stephenson data is available in the EBI database under accession code E-MTAB-10026 (https://www.ebi.ac.uk/biostudies/arrayexpress/studies/E-MTAB-10026).
The Su data is available in the EBI database under accession code E-MTAB-9357 (https://www.ebi.ac.uk/biostudies/arrayexpress/studies/E-MTAB-9357).
The Thompson data is available in the GEO database under accession code GSE166992 (https://www.ncbi.nlm.nih.gov/geo/query/acc.cgi?acc=GSE166992).
The Unterman data is available in the GEO database under accession code GSE155224 (https://www.ncbi.nlm.nih.gov/geo/query/acc.cgi?acc=GSE155224).
The Wilk data is available in the GEO database under accession code GSE174072 (https://www.ncbi.nlm.nih.gov/geo/query/acc.cgi?acc=GSE174072).
The Yao data is available in the GEO database under accession code GSE154567 (https://www.ncbi.nlm.nih.gov/geo/query/acc.cgi?acc=GSE154567).
The Zhao data is available at Figshare (https://figshare.com/articles/dataset/seu_obj_h5ad/16922467).
The Zhu data is available at the CNGB database under project code CNP0001102 (https://db.cngb.org/search/project/CNP0001102/).

The COVID-19 IMC data. The COMBAT data used in this study is available in the EGA database under accession code EGAS00001005493 (https://ega-archive.org/studies/EGAS00001005493). The Geanon data is available at FlowRepository under the accession code FR-FCM-Z2XA (http://flowrepository.org/id/FR-FCM-Z2XA).

The COVID-19 IMC data.
The Rendeiro data is available in Zenodo under the accession code zenodo.4110560, zenodo.4139443 and zenodo.4637034 (https://zenodo.org/record/4110560,
https://zenodo.org/record/4139443,
https://zenodo.org/record/4637034,
)

Human research participants

Policy information about studies involving human research participants and Sex and Gender in Research.

Reporting on sex and gender	n/a
Population characteristics	n/a
Recruitment	n/a
Ethics oversight	n/a

Note that full information on the approval of the study protocol must also be provided in the manuscript.

Field-specific reporting

Please select the one below that is the best fit for your research. If you are not sure, read the appropriate sections before making your selection.

- [x] Life sciences
- [ ] Behavioural & social sciences
- [ ] Ecological, evolutionary & environmental sciences

For a reference copy of the document with all sections, see nature.com/documents/nr-reporting-summary-flat.pdf

Life sciences study design

All studies must disclose on these points even when the disclosure is negative.

Sample size	We evaluated our method by using a data collection of scRNA-seq data with 20 studies, a CyTOF data collection, a IMC data collection. We selected these datasets as they represent a wide ranges of data characteristics of single-cell datasets, including different number of cells, number of cell types, number of features, technologies and platforms. Therefore, the datasets are sufficient to demonstrate the accurate and robust performance of our method.
Data exclusions	For Ren et al. COVID-19 dataset, we excluded the cells that are collected using FASC-sorted technology since we focus on data integration of non FACS-sorted COVID-19 PBMC or whole blood samples data.
Replication	To ensure robustness and reproducibility of the findings, we ensured that our results are robust when random subsampling of the data was generated; method are demonstrated via datasets generated by different technologies and scale; classification task was performed via

	multiple repeated cross-validation.
Randomization	Our study does not involve allocating samples to experimental groups.
Blinding	Our study does not involve group allocation that requires blinding.

Reporting for specific materials, systems and methods

We require information from authors about some types of materials, experimental systems and methods used in many studies. Here, indicate whether each material, system or method listed is relevant to your study. If you are not sure if a list item applies to your research, read the appropriate section before selecting a response.

Materials & experimental systems

n/a	Involved in the study	
☒	☐	Antibodies
☒	☐	Eukaryotic cell lines
☒	☐	Palaeontology and archaeology
☒	☐	Animals and other organisms
☒	☐	Clinical data
☒	☐	Dual use research of concern

Methods

n/a	Involved in the study	
☒	☐	ChIP-seq
☒	☐	Flow cytometry
☒	☐	MRI-based neuroimaging
